# Biostable Shape Memory Polymer Foams for Smart Biomaterial Applications

**DOI:** 10.3390/polym13234084

**Published:** 2021-11-24

**Authors:** Anand Utpal Vakil, Natalie Marie Petryk, Ellen Shepherd, Mary Beth B. Monroe

**Affiliations:** Department of Biomedical and Chemical Engineering, Syracuse Biomaterials Institute, and BioInspired Syracuse: Institute for Material and Living Systems, Syracuse University, Syracuse, NY 13244, USA; auvakil@syr.edu (A.U.V.); nmpetryk@syr.edu (N.M.P.); eshepher@syr.edu (E.S.)

**Keywords:** shape memory polymers, polyurethanes, oxidation, degradation, biostable, foams

## Abstract

Polyurethane foams provide a wide range of applications as a biomaterial system due to the ability to tune their physical, chemical, and biological properties to meet the requirements of the intended applications. Another key parameter that determines the usability of this biomaterial is its degradability under body conditions. Several current approaches focus on slowing the degradation rate for applications that require the implant to be present for a longer time frame (over 100 days). Here, biostable shape memory polymer (SMP) foams were synthesized with added ether-containing monomers to tune the degradation rates. The physical, thermal and shape memory properties of these foams were characterized along with their cytocompatibility and blood interactions. Degradation profiles were assessed in vitro in oxidative (3% H_2_O_2_; real-time) and hydrolytic media (0.1 M NaOH; accelerated) at 37 °C. The resulting foams had tunable degradation rates, with up 15% mass remaining after 108 days, and controlled erosion profiles. These easy-to-use, shape-filling SMP foams have the potential for various biomaterial applications where longer-term stability without the need for implant removal is desired.

## 1. Introduction

Shape memory polymers (SMPs) are smart materials with many potential biomedical applications. SMPs can be prepared in a primary/original shape, deformed into a temporary shape upon exposure to an external stimulus, and stored in this temporary shape once the stimulus is removed. The external stimulus can be temperature, light, pH, electrical stimulus, or a magnetic field. Upon re-exposure to the stimulus, the shape memory effect can be triggered to recover the material back to its original shape.

Based on the application, biomaterials require varying degrees of biodegradability, tissue integration, cell and blood interactions, nutrient transfer, space-filling ability, and clinical functionality. Polyurethane SMPs have been extensively employed as biomaterial scaffolds in vascular applications, [1] drug delivery, [2] and tissue engineering due to their excellent tunable mechanical properties, [3] high cytocompatibility and biocompatibility, [4,5] and the ability to tune degradation rates to match application requirements [6,7].

Biodegradation affects cell infiltration, vascular in-growth, and neo-tissue formation to allow successful integration of host tissue with biomaterials at the implant location. Biodegradation can occur via three major mechanisms: oxidation, hydrolysis, and enzymatic degradation [8]. Some applications, such as degradable sutures, require a fast degradation rate, while others require biostable scaffolds that remain in the body over long time frames. Polyurethane SMPs present an ideal system for controlling degradation rates by selectively incorporating oxidatively, hydrolytically, and/or enzymatically responsive groups. The ability to control architecture changes with shape memory properties while tuning degradation profiles presents several potential benefits for healing, and previous research in this area is rich [9,10,11].

Within the large field of polyurethane SMPs, a crosslinked, amorphous polyurethane SMP foam system has been used for vascular occlusion applications, such as aneurysm filling [12,13], peripheral vascular disease [14], and hemorrhage control [15]. These materials are highly tunable, with prior work focusing on altering shape memory profiles, [16] pore structure [17], and/or toughness [18] and on incorporation of functional motifs to enable in vivo imaging [19,20] or infection control [21]. In vitro degradation characterization of SMP foams showed that they are hydrolytically stable, but that they degrade via oxidation [7]. Degradation was attributed to tertiary amines in the polyol crosslinkers that are used to form the polyurethane network. In a rabbit elastase aneurysm model, Herting et al. found that the materials underwent ~97% mass loss by 90 days using cross-sectional histological images [22].

Based on these findings, several subsequent studies have focused on improving the biostability of this valuable biomaterial system. Hasan et al. replaced the tertiary amine-containing monomers in the SMP foams with glycerol and hexanetriol. These foams were highly stable, with <10% mass loss over 45 days in accelerated hydrolytic and oxidation medias (0.1 M NaOH and 50% H_2_O_2_, respectively) [23]. However, their shape recovery profiles were significantly slower than the original SMP foams, with 100% volume recovery achieved after ~40 min in 50 °C water vs. full recovery in <10 min in 37 °C water in the control foams. This property would limit their ability to be actuated upon implantation at body temperature in future applications.

Weems et al. focused on improving the biostability of shape memory polyurethanes by incorporating isocyanurate-containing alcohols [24]. This approach resulted in increased biostability and delayed degradation. While tested in an accelerated oxidative degradation media (20% H_2_O_2_ catalyzed by 0.1 M CoCl_2_), SMP films had more than 80% mass remaining after 100 days and porous SMP foams had close to 75% mass remaining after 40 days. This is a promising approach to significantly increasing biostability; however, the eventual degradation byproduct of these polymers may contain small molecules like cyclic isocyanurates, whose cytocompatibility has not been determined. Additionally, materials with intermediate degradation rates may be required.

In a separate approach, Weems et al. achieved a reduced degradation rate by incorporating small molecule antioxidants into the foams to form an SMP composite [25]. The microparticles were physically mixed within the polymer solution; thus, this approach could result in the antioxidant-loaded microparticles leaching out of the polymer system to alter the scaffold biostability over time. In most of the composite formulations, the antioxidant payloads were released during the initial cleaning procedure. The composite that did have a well-retained antioxidant content after washing underwent complete release of the antioxidant during the first three days of the degradation study, and thus did not significantly alter the long-term degradation profile.

While these SMP foams have been widely employed in embolic applications, none of the prior studies characterized blood interactions following modifications, which are highly dependent on material chemistry. Additionally, there may be benefits to more moderate increases in biostability or in altering the physical erosion profiles of SMP foams, such as in load-bearing applications where bulk device failure may be detrimental to healing. To that end, we synthesized polyurethane SMP foams that were modified with ether linkages using diethylene glycol (DEG) or triethylene glycol (TEG) to extend their biostability relative to control foams. We characterized the ability to tune the rate of degradation while maintaining other properties, such as pore size and volume recovery rates, the physical erosion profiles, and cell and blood interactions in the resulting ether-containing foams. In the long-term, these foams could provide an option for biomaterial implants with controlled degradation after implantation to maintain scaffold properties over longer time frames and to eliminate the need for implant removal.

## 2. Materials and Methods

Materials: Hexamethylene diisocyante (HDI), diethylene glycol (DEG), triethylene glycol (TEG), N,N,N′,N′-tetrakis-(2-hydroxypropyl)-ethylene diamine (HPED), triethanol amine (TEA), hydrogen peroxide (H_2_O_2_, Certified ACS, 30%), sodium hydroxide (NaOH), and ethanol (reagent alcohol) were purchased as used as received from Fisher Scientific (Waltham, MA, USA). All chemicals were purchased at reagent grade unless specified. Catalysts (T-131 and BL-22) and surfactant (EP-H-190) were used as received from Evonik Corporation (Essen, Germany).

Foam Synthesis: Polyurethane foams were synthesized in a two-part process by first preparing an isocyanate (NCO) pre-mix that contained 35 equivalents of hydroxyl groups provided by varying ratios of HPED, TEA, DEG, and TEG, and 100 equivalents of isocyanates. The pre-polymer was formed by crosslinking the NCO pre-mix at 50 °C for 48 h. Surfactant (EP-H-190) was added to the pre-mix after 48 h. The NCO pre-mix was allowed to cool down to room temperature while the hydroxyl pre-mix was made. The hydroxyl (OH) mix contained the remaining hydroxyls to balance the NCO groups, deionized (DI) water as a chemical blowing agent, and catalysts (T-131-tin based gelling catalyst) and BL-22-amine based blowing catalyst). The hydroxyl components were mixed at 3500 rpm for 30 s. The required amount of catalysts were added to the hydroxyl contents and mixed at 3500 rpm for 30 s. The final hydroxyl mix was added to the isocyanate pre-mix and mixed at 1800 rpm for 5 s and poured into a large mold to form a gas-blown foam in an isothermal oven maintained at 50 °C. All mixing was carried out in a high-speed mixer (Flacktek, Landrum, SC, USA). The overall hydroxyl to isocyanate ratio in the foam was maintained at 1.04 to ensure the complete reaction of isocyanates during the synthesis. Synthesized foam compositions are shown in Table 1.

Foam Pore Analysis: Foam slices (n = 3, ~1 cm^2^) were cut parallel and perpendicular to the foam rise direction. Each piece was coated with gold using a high vacuum sputter coater (Denton, Moorestown, NJ, USA) at 100 mTorr for 45 s to form a consistent and a stable coating. Pore structures were characterized via a JEOL JSM 5600 scanning electron microscope (SEM; JEOL USA, Peabody, MA, USA) at 35× magnification under 10 kV high vacuum. The micrographs were analyzed via ImageJ (National Institutes of Health, Bethesda, MD, USA) to quantify pore diameters.

Density: Cube samples (n = 3, ~1 cm^3^) were cut via a hot wire cutter, (Proxxon Thermocut 115/E, Hickory, NC, USA). Dimensions and weights were measured to obtain foam densities.

Mechanical Testing: Dogbone punches were cut from each foam (n = 3) according to the ASTM D638 (scaled down by a factor of 4; length: 6.25 mm, width: ~1.5 mm). The thickness of each piece was measured prior to testing. Samples were tested in both dry and wet conditions. To test the samples in wet conditions, they were placed in DI water at 50 °C for 5 min and patted dry prior to analysis. Based on thermal and swelling analysis, this time frame/temperature provided equilibrium water absorption to ensure sample wetting. Samples (n = 3) were stretched in a tensile tester via a 24 N load cell at a rate of 2 mm/min until failure to measure elastic modulus, elongation at break, and ultimate tensile strength.

Thermal analysis: Glass transition temperature (Tg) was measured for each sample (n = 3, 3–5 mg) using a Q200 differential scanning calorimeter (DSC, TA instruments, New Castle, DE, USA) in both dry and wet (plasticized) conditions. Samples were placed in t-zero aluminum pans, equilibrated at −40 °C, heated to 120 °C at 10 °C/min, kept isothermally for 2 min, cooled to −40 °C at 10 °C/min, and heated back to 120 °C at 10 °C/min. Dry Tg was measured was measured as the half-height transition temperature during the second heating cycle. To measure wet Tg, samples were plasticized by placing in DI water at 50 °C for 10 min, pressed dry, and placed in t-zero aluminum pans with hermetic lids. A pin hole was pierced on the hermetic lid to allow water to escape during the heating cycle. Samples were equilibrated at −60 °C and heated to 80 °C at 10 °C/min. Wet Tg was measured as the half-height transition temperature during the single heating cycle.

Shape Memory Behavior: Volume expansion was used to quantify shape memory behavior. Cylindrical foam samples (1 cm long, 8 mm diameter) were cut, cleaned in DI water and 70% ethanol, and dried under vacuum for 24 h prior to testing. Each sample was heated to 100 °C for 10 min to allow softening, and the diameter was recorded using digital calipers prior to manual crimping in a radial compression crimper (Blockwise Engineering, Tempe, AZ, USA). After cooling to room temperature, the final crimped sample diameter and length were recorded, samples were placed in scintillation vial in a desiccator for 24 h and fixed on a 300 µm Nitinol wire to allow for complete shape setting and relaxation to occur. After 24 h, the foam’s initial diameter and length were measured, and samples were placed in a DI water bath set at 37 °C and allowed to expand for 5 min. Images were captured every 3 s to observe changes in diameter over time (t) and generate a volume recovery profile. Images were analyzed using ImageJ and volume recovery was measured as:

(1) $$\%\;\mathrm{Volume}\;\mathrm{Recovery}=\frac{\mathrm{Sample}\;\mathrm{Diameter}\left(t\right)\;\times \;\mathrm{Sample}\;\mathrm{Length}\left(t\right)\;}{\mathrm{Initial}\;\mathrm{Diameter}\;\left(d1\right)\;\times \;\mathrm{Initial}\;\mathrm{Length}\;\left(l1\right)\;}\;\times \;100$$

Change in volume vs. time was plotted over the expansion time frame.

Spectroscopic Analysis: Surface chemistry was characterized on thin slices of cleaned foam pieces using a Nicolet i70 Attenuate total reflectance (ATR)-Fourier transform infrared (FTIR) Spectrometer (Fisher Scientific, Waltham, MA, USA) at 0.8 cm^−1^ resolution using OMNIC software (Fisher Scientific, Waltham, MA, USA). Incorporation of ethers into polyurethane foams was confirmed by the presence of peaks corresponding to the C-O of the ether group at ~1090 and ~1050 cm^−1^ and the carbonyl of urethane at ~1688 cm^−1^.

Degradation Analysis: Cylindrical foams (n = 8, 8 mm diameter, 1 cm height) were washed and dried, and initial masses were obtained using a gravimetric scale. Samples were placed in 3% H_2_O_2_ (real-time oxidative degradation media) or in 0.1 M NaOH (accelerated hydrolytic degradation media) at 37 °C with regular media changes. At selected time points, samples were washed with ethanol and dried under vacuum for 24 h. After drying, samples were imaged using a camera, and masses were measured (n = 5). A thin slice was cut from a sacrificial set of foams (n = 3) and used to measure pore morphology (SEM), Tg (DSC), and surface chemistry (FTIR) as described above.

Cytocompatibility: Sample cytocompatibility was tested using 3T3 Swiss mouse fibroblasts (ATCC-CCL92; ATCC, Manassas, VA, USA). Cells were cultured with Dulbecco’s modified Eagle’s medium (DMEM, high glucose GlutaMAX), supplemented with 10% heat-inactivated fetal bovine serum (FBS) and 1% penicillin-streptomycin (P/S, Gibco) at 37 °C/5% CO_2_. Cells from passage 11 were used after three days of culture. Cells were seeded onto a 24-well tissue culture polystyrene plate at 10,000 cells/well and incubated for 24 h at 37 °C/5% CO_2_ for 24 h. Samples were cleaned using water, 20% Contrad solution, and isopropyl alcohol, and then soaked in 1× PBS overnight prior to testing to leach out any alcohol. Samples (n = 3) were placed in each well along with positive controls (media-only with cells, n = 3), and negative controls (media-only with no cells). Samples were incubated with cells, and viability was assessed after 24 h using a Live/Dead assay (ThermoFisher Scientific, Waltham, MA, USA). Briefly, cells were stained with green fluorescent calcein-AM (live cells) and red-fluorescent ethidium homodimer-1 (dead cells) for 15 min at room temperature while protecting from light. Cells were imaged using an inverted microscope (Leica, DMI6000) at 10× magnification to determine the number of live (green) and dead (red) cells. Three images were captured for each sample. Cell viability of each sample (x) was measured as:

(2) $$\mathrm{Cell}\;\mathrm{Viability}\;\left(x\right)=\frac{Live\;Cells}{Total\;number\;of\;cells}\times 100\%$$

Blood Interactions: Porcine blood (Lampire Biological Laboratories, Pipersville, PA, USA) anticoagulated with Na-Citrate upon collection was stored at 4 °C for up to 3 weeks from the bleed date, according to supplier guidelines. Control, 30% DEG, and 30% TEG foams were washed and dried prior to characterization in all studies. QuikClot Combat Gauze was included as a clinical control. Blood absorption was analyzed by weighing dry samples (n = 3; ~50 mg) and incubating them in blood at 37 °C. Samples were weighed at 24 h, and blood absorption was calculated as:

(3) $$\%\;Absorbed=\frac{W_{b}-W_{d}}{W_{d}}\times 100\%$$

where *W_b_* is the mass of the sample in blood and *W_d_* is the dry mass.

Platelet attachment was measured via LDH cytotoxicity assay kit (Cayman Chemical, Ann Arbor, MI, USA). Platelet-rich plasma (PRP) was collected by centrifuging whole blood at 3000 rpm for 15 min to obtain a standard curve. PRP was diluted with PBS to obtain multiple concentrations (100, 50, 25, 12.5, and 6%) to generate a standard. Hemocytometer counts were acquired at each PRP concentration (n = 4) to quantify the standard values. SMP foams (n = 4) were cut to equal surface area and placed in individual wells in a 24-well plate. Gauze was used as a clinical control. One milliliter of blood was added to each well and the soaked samples were incubated at 37 °C for 30 min. PBS was used to wash out any unattached platelets. Samples were then added to wells on a separate plate containing 1 mL PBS and 100 µL of 10% Triton X-100 to lyse unattached platelets. Supernatant (100 µL) from each well was added to wells on a separate 96 well plate along with 100 µL of LDH reaction solution. The mixture was incubated at 37 °C for 30 min on an orbital shaker. Microplate reader was then used to obtain absorbance values from each sample at 490 nm.

Samples that were washed with PBS to remove unattached platelets were then imaged via SEM to observe activity states and platelet activation. Prior to imaging, samples were soaked in 2% glutaraldehyde solution (Electron Microscopy Sciences, Hartfield, PA, USA) to fix them and later dehydrated. To achieve complete dehydration, samples were soaked in a series of ethanol concentrations: (1) 30 min in 50% ethanol, (2) 30 min in 70% ethanol, (3) 30 min in 95% ethanol and finally (4) 30 min in 95% ethanol. Post dehydration samples were dried overnight in a vacuum oven at 50 °C and −30 inches Hg. SEM analysis was performed using JEOL NeoScope JSM-5600 (JEOL USA, Peabody, MA, USA) operated at 10 kV. Images were captured at regions of interest at 1000× and 5000× magnification. These images were later analyzed via ImageJ assess platelet aggregations and activation (morphology change).

The time required for coagulation was measured for each sample (n = 4) by placing them in 1.5 mL microcentrifuge tubes and exposing them to blood. One tube was maintained as a negative control with no sample. Samples were weighed and cut to have the same surface area throughout. Blood was brought to room temperature and the Na-citrate anticoagulant was reversed by adding 1 M CaCl_2_ solution to obtain a net 0.01 M CaCl_2_ solution. Then, 50 µL of this blood was added to each sample tube. The clotting process was stopped at each time point (every 6 min over 30 min) by adding 1 mL DI water to each tube to lyse the free red blood cells. These tubes were centrifuged at 2300 rpm for 15 min, inverted, and images were captured using a digital camera (AKASO V50 Pro Native, AKASO, Frederick, MD, USA). The relative amount of hemoglobin released at each time point was determined by adding 200 µL of the lysate from each tube to a 96-well plate and measuring the absorbance at 540 nm using a BioTek Synergy 2 Multimode Microplate Reader (Winooski, VT, USA).

Statistical Analysis: Measurements are presented as mean ± standard deviations. Student’s *t*-tests were performed to determine differences between ether foams and controls. Statistical significance was taken as *p* < 0.05.

## 3. Results

### 3.1. Structural Properties

**Figure 1:**
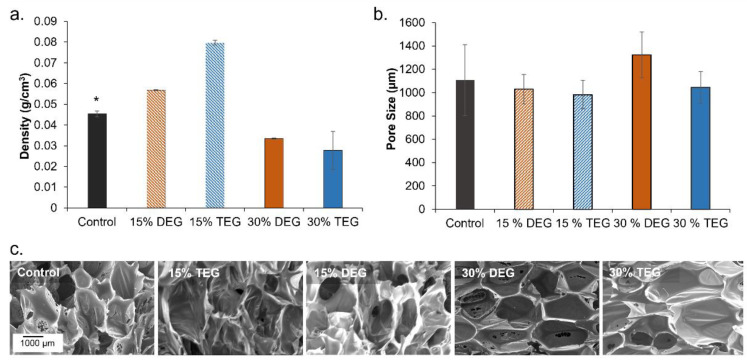
Structural properties of shape memory polymer foams. (**a**) The density of foams (n = 3), (**b**) average pore size of foams (n = 6) measured using SEM images on samples cut parallel and perpendicular to foam rise, and (**c**) representative micrographs of pore morphology. Scale bar of 1000 µm applies to all images. Mean ± standard deviation displayed in all panels. * *p* < 0.05 relative to all other foams.

Low density polyurethane foams were synthesized with a target density below 0.08 g/cm^3^, Figure 1a. General increases in density were observed with the introduction of lower amounts (15%) of ether-containing monomers, while general decreases in density were observed in higher ether content foams (30%). Pore sizes for each foam were targeted to be between 1000 and 1400 µm to ensure comparable properties to the control. 30% DEG foams have the largest pore size of 1323 µm (vs. control foams: 1151 µm), Figure 1b. In the SEM images, Figure 1c, 15% DEG and TEG foams appear to have thicker walls, which corresponds with their increased density. In addition to the higher pore size in 30% DEG foams, evidence of pore opening (pinholes in pore walls) can be observed in the 30% DEG and TEG foam SEM images, which resulted in lower density relative to the control foam.

### 3.2. Thermal Properties

**Figure 2:**
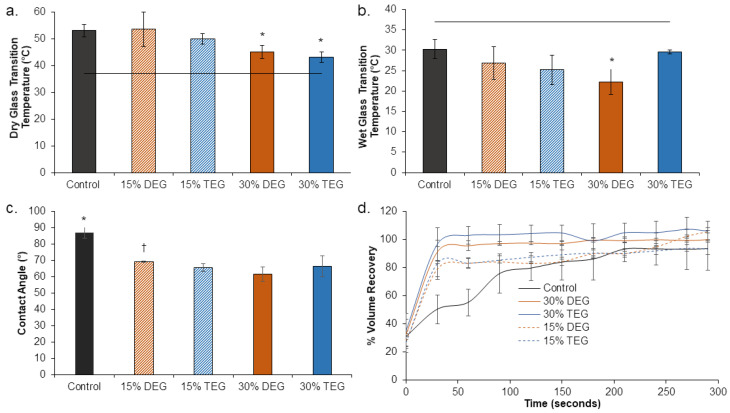
Thermal, shape memory, and hydrophilicity properties of SMP foams. (**a**) Dry glass transition temperature (n = 3, * *p* < 0.05 relative to control), (**b**) wet glass transition temperature (n = 3, * *p* < 0.05 relative to control), horizontal line is provided as a reference for body temperature (37 °C) in (**a**,**b**). (**c**) contact angle (n = 5, * *p* < 0.05 relative to all other samples, † *p* < 0.05 relative to 15% TEG and 30% DEG samples), and (**d**) volume recovery of samples (n = 3) in deionized water at 37 °C. Mean ± standard deviation displayed in all panels.

Highly crosslinked amorphous networks were formed using polyol crosslinkers with three (TEA) and four (HPED) hydroxyl groups, along with short-chain diol monomers (DEG and TEG), which was indicated by the absence of melting peaks in the DSC traces (Figure S1 in Supplementary Materials). As seen in Figure 2a, all foams had dry T_g_’s above 40 °C, which enables stable storage of foams at room temperature (~22 °C) without premature shape memory actuation. The target wet glass transition temperature below 37 °C was also obtained in all foams, Figure 2b, which enables actuation of shape memory behavior upon implantation and exposure to water present in the body via water-induced plasticization of the SMPs.

### 3.3. Hydrophilicity and Shape Memory

The contact angle was measured on each formulation in bulk film form to compare the difference in water interactions between the foams. Control films had the highest contact angle (87°) and the inclusion of DEG and TEG increased hydrophilicity, as evidenced by decreased contact angles (down to 63° for TEG and 70° for DEG), as shown in Figure 2c. Shape recovery profiles of samples were evaluated to indicate their capability to return from their secondary, compressed shape to their original, expanded shape after implantation and exposure to water in the body, Figure 2d. All foams expanded back to 100% of their original volume within ~200 s. In general, volume expansion profiles were similar, but the 15% DEG and TEG foams had faster expansion in the first 30 s, and the 30% DEG and TEG foams had slower expansion in the first 60 s relative to the control.

### 3.4. Tensile Testing

The addition of ether-containing diol monomers resulted in an overall reduction of elastic modulus and an increase in maximum elongation in the wet and dry states compared to controls, Table 2. The highest reduction in modulus relative to the control (22×) was observed in 30% TEG foams, which corresponds with the highest increase (8×) in elongation at break. All foams had a reduction in modulus and the corresponding increase in elongation after undergoing water-induced plasticization in DI water at 50 °C for 5 min. The wet foam mechanical properties were overall more similar between formulations.

### 3.5. Degradation Analysis

#### 3.5.1. Mass Loss and Physical Erosion

**Figure 3:**
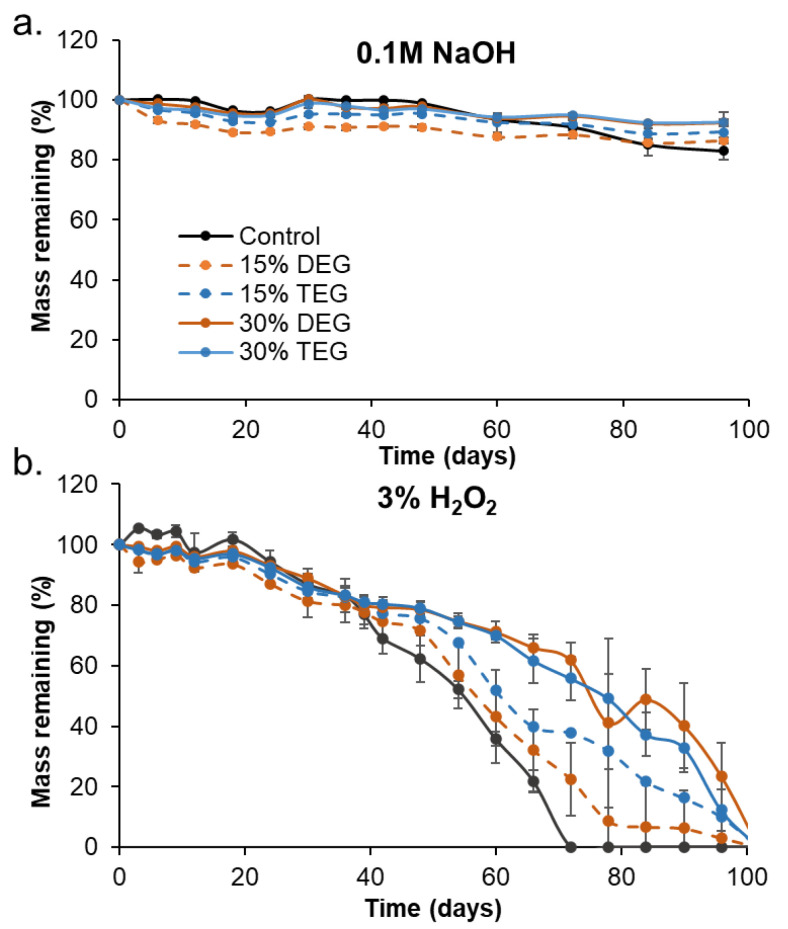
Mass loss profiles of samples as a function of time in (**a**) accelerated hydrolytic degradation media, 0.1 M NaOH, and (**b**) oxidative degradation media, 3% H_2_O_2_. n = 5, mean ± standard deviation displayed. Legend provided in (**a**) applies to both charts.

**Figure 4:**
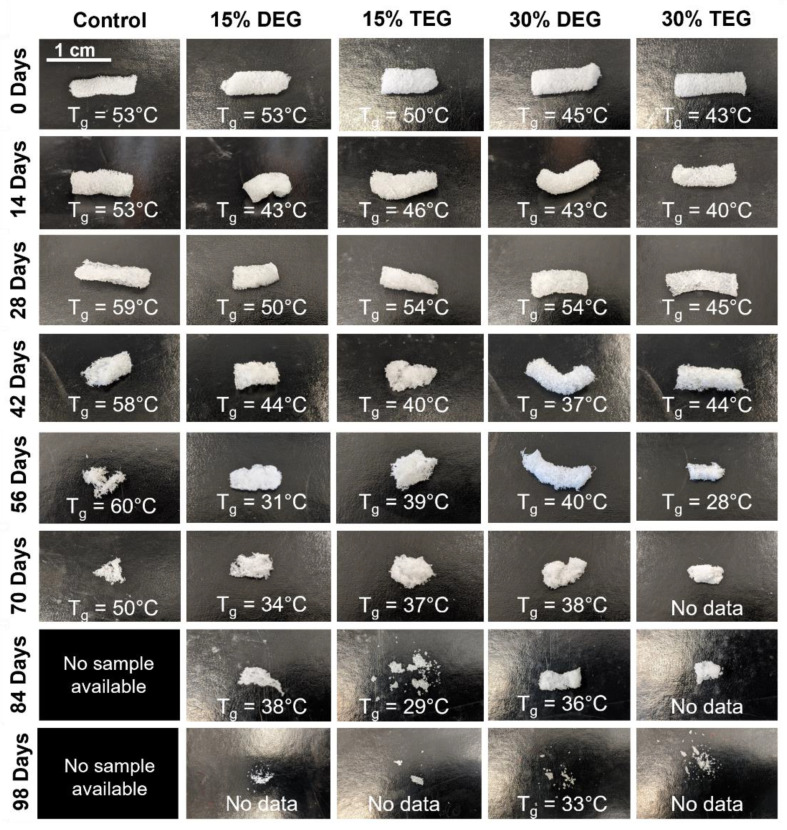
Physical erosion profile of samples degrading in oxidative degradation media, 3% H_2_O_2_ along with respective T_g_’s measured at each time point. Scale bar applies to all images.

**Figure 5:**
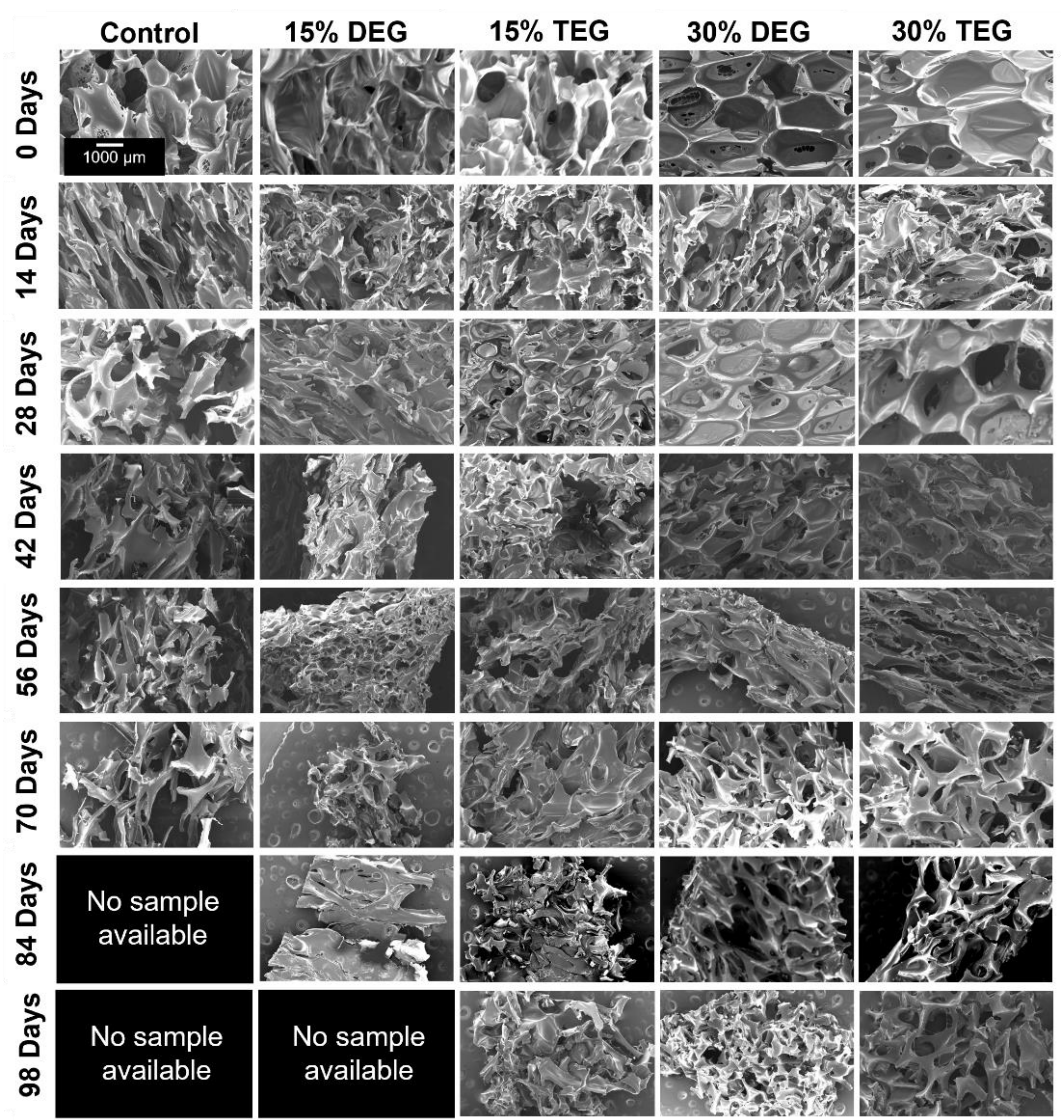
SEM micrographs depicting the overall pore morphology observed during degradation in oxidative media, 3% H_2_O_2_, over 98 days. Scale bar of 1000 µm applies to all images.

All foams remained stable in accelerated hydrolytic media (0.1 M NaOH), with less than 10% mass loss over 98 days, Figure 3a. In the oxidative media (3% H_2_O_2_) foams had comparable, approximately linear mass loss rates over the first 40 days, Figure 3b. After that, control foams began to degrade more quickly, and they underwent bulk erosion and started breaking into smaller pieces by ~56 days, Figure 4. Amongst the ether-containing foams, 30% DEG foams had the slowest degradation rate and had 5% mass remaining after 105 days, followed by 30% TEG foams, which fully degraded in 98 days. The ether-containing foams appeared to undergo surface erosion, maintaining their bulk geometries over >80 days, Figure 4 and Figure 5.

#### 3.5.2. Thermal Analysis

As degradation proceeded, thermal analysis was performed to measure T_g_ as an indication of network crosslink density over time, Figure 4. This data can be used as an indication of whether the foams underwent surface or bulk degradation, where surface degradation would indicate that the polymer network and crosslink density remain intact during material breakdown. Interestingly, despite their observed physical bulk erosion, Figure 4, control foams retained their T_g_ (~50–60 °C) throughout the entire degradation process. Due to complete sample degradation, no images could be obtained of control foams past 70 days. All ether-containing foams retained their T_g_’s until ~56 days, after which there was an observed decrease in T_g_. Thus, surface degradation likely occurred throughout most of the degradation process, as is expected for oxidative degradation, due to the high reactivity of reactive oxygen species.

#### 3.5.3. Pore Morphology

SEM was used to analyze pore morphology every two weeks, as shown in Figure 5. Control foams began losing their porous structure by 14 days and underwent significant strut breaking by 28 days. Total pore collapse was observed in control foams by 42 days. Among the ether-containing foams, 30% DEG and 30% TEG generally maintained their pore morphology while shrinking over time, with some strut breakage at ~70 days and collapse at ~98 days. Material shrinkage can also be seen in 15% DEG and TEG foams, with maintained visible pores over ~70 days. Due to complete sample degradation, no images could be obtained of control foams past 70 days or of 15% DEG foams past 84 days.

#### 3.5.4. Spectroscopic Analysis

**Figure 6:**
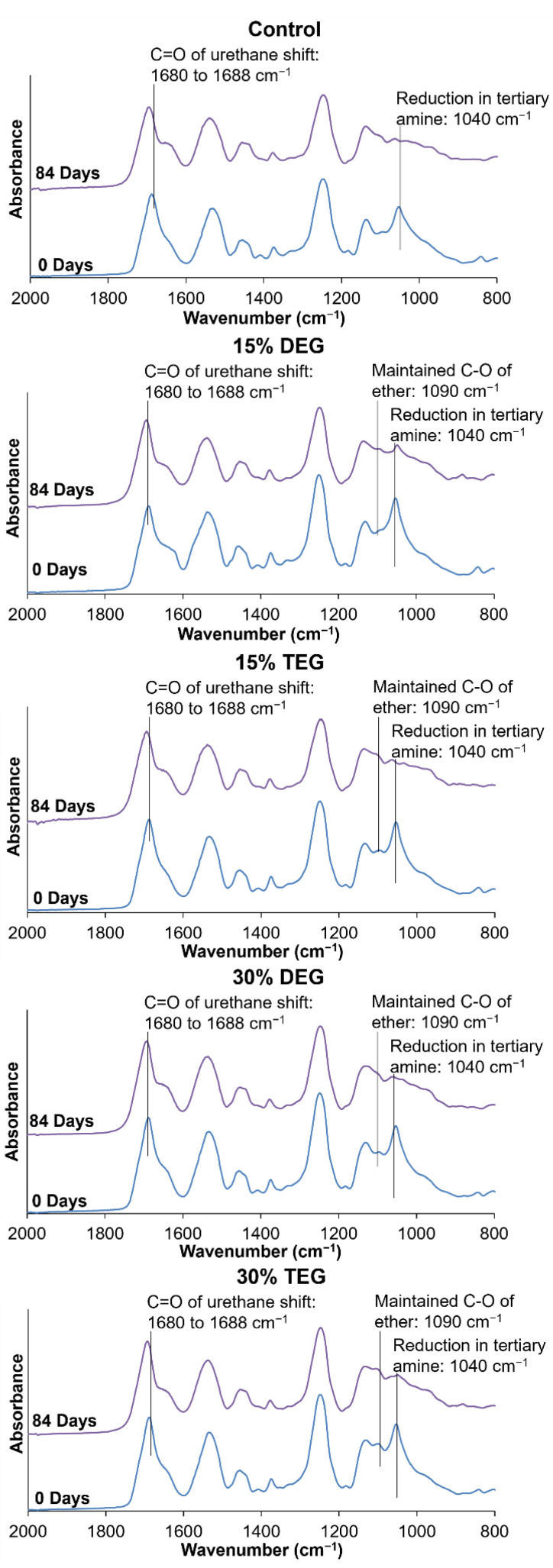
FTIR spectroscopic analysis of samples during degradation in oxidative media, 3% H_2_O_2_ at 0 days (blue lines) and 84 days (purple lines).

FTIR spectra during degradation in 3% H_2_O_2_ revealed a shift in the urethane peak from 1680 cm^−1^ to 1688 cm^−1^ and a reduction in tertiary amine peaks of HPED and TEA at 1050 cm^−1^, which has been previously observed, Figure 6 [7]. As the tertiary amine peak in the ether-containing foams is reduced, the ether peak at ~1090 cm^−1^ become more apparent, indicating that the ether groups remain stable during degradation. There is no visible evidence of ether crosslinking (branched ether peak at ~1174 cm^−1^) during degradation in the FTIR spectra [26].

### 3.6. Cell and Blood Interactions

**Figure 7:**
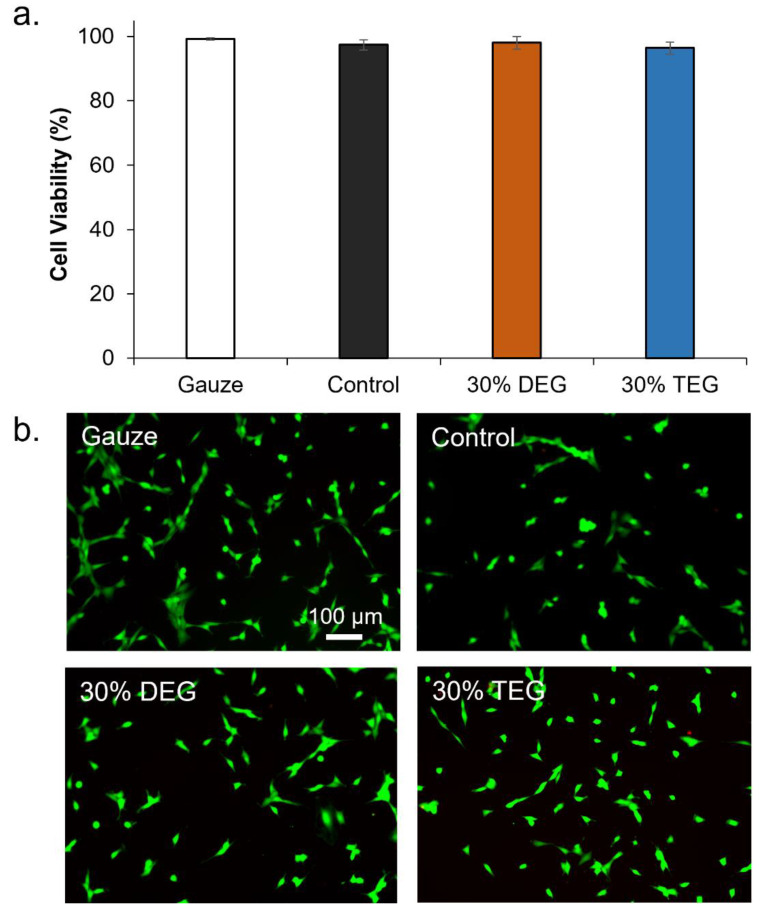
Cytocompatibility of SMP foams. (**a**) 3T3 mouse fibroblast viability over 24 h (n = 3). (**b**) Representative live/dead cell images were used to calculate viability. Live cells were stained green and dead cells were stained red. Scale bar applies to all images.

**Figure 8:**
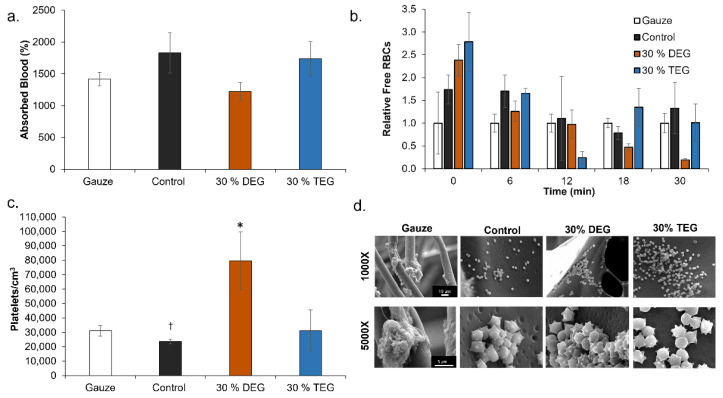
Cell and blood interactions with SMP foams. (**a**) Whole porcine blood absorption after 24 h of incubation (n = 3). (**b**) Average blood coagulation profiles represented free RBCs relative to clinical control (gauze) over 30 min (n = 4). * *p* <0.05 relative to gauze. (**c**) Platelet attachment to sample surfaces (n = 3, * *p* < 0.05 relative to all samples, ^†^ *p* < 0.05 relative to gauze). Mean ± standard deviation displayed in all panels. (**d**) SEM micrographs of attached platelets. Scale bars are shown in Gauze column apply to all other images in each row.

Since 15% DEG and 15% TEG had a faster degradation rate compared to 30% DEG and 30% TEG samples, cell and blood interactions were studied exclusively for the foams containing 30% ether linkages along with control foams and a clinical control (QuickClot gauze). Cell viability was confirmed to be ~100% for all samples after 24 h of incubation, Figure 7a. Images of live/dead cells can be seen in Figure 7b. Live cells are stained green and dead cells are stained red. As seen in Figure 8a control foams absorbed the highest amount of blood among the tested samples, and all materials absorbed between 100 and 200% of their dry weight in blood. In the coagulation study, the amount of free RBCs was higher in SMP foams relative to gauze at 0 min. However, comparable coagulation profiles were observed by 6 min Figure 8b. At 18 min and beyond, 30% DEG had the lowest number of free RBCs amongst all test samples, indicating a higher clotting capability. Images of lysates can be seen in Supplementary Materials Figure S2. Platelet attachment was quantified after incubation of samples in platelet-rich plasma. As shown in Figure 8c, maximum platelet attachment was observed on 30% DEG, followed by gauze and 30% TEG with comparable platelet numbers that were approximately half that of 30% DEG. Control foams had the lowest number of attached platelets. These results correlate the platelet aggregation and activation visualized using SEM micrographs, Figure 8d. The gauze clinical control had aggregated platelets with evidence of thrombus formation. All three SMP foams showed evidence of platelet activation (small protrusions on platelet surfaces) and aggregation, and imaged platelet densities correspond with the numbers quantified using the LDH assay.

## 4. Discussion

Overall, it was observed that adding ether-containing monomers, DEG and TEG, resulted in increased pore interconnectivity and reduction in T_g_ compared to control foams. We hypothesize that the increased hydrophilicity of DEG and TEG enabled increased interactions between the monomers in the pre-polymer and the chemical blowing agent (water) and/or the surfactant, which resulted in pore opening in the 30% DEG and TEG foams. This phenomenon could be advantageous in applications that require increased interconnectivity without relying on physical or mechanical modifications like mechanical reticulation, [17] plasma treatment, [27], and/or the addition of physical blowing agents.

TEG-containing foams had slightly lower T_g_’s compared to corollary DEG-containing foams and increasing TEG and DEG content induced further decreases in T_g_. Reduction in foam T_g_ is attributed to increased hydrophilicity and flexibility of DEG and TEG, which corresponds with the contact angle measurements. Additionally, replacing the tri-functional TEA with difunctional DEG or TEG theoretically reduces foam crosslink density, which would result in lower T_g_. However, all foams had dry T_g_ well above room temperature, which would enable their stable storage in the secondary shape. Exposure to water at 37 °C results in a reduction in T_g_ due to plasticization by water molecules penetrating the inner structure of the foams. This reduced T_g_ aids in rapid volume recovery once implanted in the body and exposed to water in body temperature blood.

The factors that determine the volume recovery of foams are wet T_g_, pore size, and hydrophilicity. Higher hydrophilicity (lower contact angle) allows for easier water absorption that corresponds to faster plasticization of foams, which is also indicated by a lower wet T_g_. Larger pore size can also increase water penetration speed into foams, further accelerating volume recovery. Compared to control foams, DEG and TEG foams have a higher hydrophilicity (lower contact angle) due to the addition of hydrophilic ether linkages. Among the ether-containing foams, 30% DEG and 30% TEG foams have increased hydrophilicity compared to 15% DEG and 15% TEG foams and have a correspondingly faster volume recovery expected. This increased volume recovery may be valuable in rapidly filling wounds during implantation.

The increased elongation at break and decreased stiffness of the ether-containing foams were expected due to the overall decreases in crosslink density and increased chain flexibility of ether linkages. The penetration of water molecules into the polymer network and interruption of hydrogen bonds allows the polymer chains to move more freely, as indicated by the overall decreased modulus and increased elongation at break of all samples in the wet conditions compared to foams tested in dry conditions. The dry measurements are important for considering material handling prior to implantation, and all materials are mechanically robust and easy to handle in the dry state. The wet measurements provide information about the material properties after implantation, which is important for matching native tissue properties. Again, all materials are mechanically within the range of soft tissues, and the differences between the ether foams and controls are reduced in the wet state [28]. In future work, the ether foams could be modified with stiffer diisocyanate species to increase the modulus if needed [16].

When incubated in an accelerated hydrolytic degradation medium containing 0.1 M NaOH, all samples remained stable, with no significant mass loss due to the lack of hydrolytically labile linkages. This result agrees with previous work on this material system that consistently shows high hydrolytic stability. [7,29] In oxidative degradation medium containing 3% H_2_O_2_, control foams physically broke apart after ~42 days, while the other formulations maintained their geometry for longer times throughout the degradation time frame. These physical changes were accompanied by an increase in the mass-loss rate in control foams. The breaking apart of control foams may be attributed to their relatively high brittleness, evidenced by the lowest elongation at break (0.17 ± 0.04 mm/mm), compared to the other formulations. During the degradation study, foams are repeatedly subjected to minor mechanical forces during the weekly washing and drying steps. Since the control foams are more brittle, they may break apart more easily and be more susceptible to bulk erosion, despite maintaining T_g_ values throughout degradation. The ether-containing foams were more flexible and less susceptible to these stresses, as demonstrated by their increased overall physical integrity throughout degradation, which translates to slower and more consistent degradation rates. The ether linkages appear to remain intact during the degradation process, as evidenced in FTIR spectra. This stability of the ether linkages could potentially contribute to their increased stability and more consistent degradation profiles.

While the overall changes in degradation rates were not hugely different, the introduction of ether linkages increased the oxidative stability by ~40% (an increase from 72 days for control to 100+ days for ether foams). Additionally, the observed surface erosion and maintenance of pore structure over longer time frames may be beneficial for graded load transfer during new tissue formation as the SMP foams degrade. Finally, the ability to tune degradation independently of thermal and shape memory properties enables easy transition to ether-containing foams to increase degradation rates without altering storage or implantation considerations.

While future studies will require more in-depth analysis of biocompatibility after implantation and cytocompatibility of degradation byproducts, the addition of TEG and DEG does not affect the cytocompatibility of SMP foams. In terms of blood absorption, the increased absorption by TEG foams compared to DEG foams is attributed to increased hydrophilicity, which increases fluid uptake. The increased absorption by control foams may be attributed to their closed pore structure, which increases blood retention compared to open pore ether foams.

Various surface characteristics, like surface charge, relative hydrophilicity, and surface roughness, can impact protein absorption and subsequent blood and/or cell interactions with biomaterials. Thus, blood interactions must be considered when making chemical changes in any biomaterial system, particularly for embolic applications. All samples had complete clotting within 12 min as seen by a reduction in free RBCs. The highest clotting at later time points (>18 min) was observed on 30% DEG foams, which corresponds with the higher platelet attachment observed on these foams, both in the quantified LDH assay and the qualitative SEM imaging. The 30% TEG foams had similar platelet attachment values to clinical gauze control, and control foams showed the lowest number of attached platelets. When visualized using SEM, gauze promoted thrombus formation within the testing time frame, while all SMP foams had aggregated and activated platelets with similar trends observed in relative platelet numbers on each surface. This result shows that incorporating ether linkages into the SMP foams enhanced platelet attachment and activation, which may translate to increased efficacy of these materials in embolic applications and provides a new tool for increasing clotting in SMP foams. These results are analyzed in a static model. Going further, we will focus on analyzing clotting capabilities using a dynamic in vitro hemorrhage model where blood is allowed to flow through the foams [30].

These smart biomaterials with increased biostability and excellent biocompatibility have a wide application in multiple tissue engineering applications. One such application involves the use as a temporary embolic device in minimally invasive medical applications that may require removal after a certain time point. The removal process can be avoided using these biomaterials.

## 5. Conclusions

A reduction in SMP foam degradation rate was achieved by incorporating ether linkages. The resulting foams maintain desired thermal properties, which allows stable storage in the secondary shape at room temperature prior to use and rapid volume recovery upon implantation. The modified foams have rapid volume recovery and increased flexibility, allowing easy implantation without premature breaks or tears. The addition of ether linkages to the foams enabled uniform surface erosion that improves retainment of scaffold integrity, which can be vital in slowly-degrading biomaterials applications. Increased clotting capabilities were seen in the 30% DEG foams that also have the slowest degradation rates. Overall, these materials could be employed in hemostatic applications and then left in place to slowly degrade during healing, eliminating risks associated with implant removal after its intended application.

## Figures and Tables

**Table 1:** Synthesized foam compositions.

Sample ID	HDI (wt%)	HPED (wt%)	TEA (wt%)	DEG (wt%)	TEG (wt%)	EPH 190 (wt%)	T-131 (wt%)	BL-22 (wt%)	Water (wt%)
Control	54.03	27.61	8.05	-	-	6.44	0.46	1.01	2.37
15% DEG	52.36	29.21	-	4.24	-	6.26	0.56	1.2	2.9
15% TEG	51.2	32.28	-	-	5.83	6.28	0.56	1.2	2.73
30% DEG	53.16	27.15	-	8.69	-	6.19	0.60	1.18	2.91
30% TEG	51.34	26.25	-	-	11.52	6.33	0.53	1.2	2.80

**Table 2:** Tensile properties of shape memory polymer foams in dry and wet conditions. n = 3, mean ± standard deviation displayed.

Sample ID	Elastic Modulus (kPa)		Maximum Elongation ε (mm/mm)	
	Dry	Wet	Dry	Wet
Control	3200 ± 1700	150 ± 20	0.17 ± 0.04	0.4 ± 0.2
15% DEG	840 ± 140	41 ± 5	0.25 ± 0.01	0.9 ± 0.5
15% TEG	140 ± 40	32 ± 6	0.46 ± 0.17	3.0 ± 1.2
30% DEG	790 ± 270	45 ± 13	0.28 ± 0.14	1.2 ± 0.2
30% TEG	170 ± 110	12 ± 3	1.25 ± 0.45	1.0 ± 0.1

## Data Availability

The data presented in this study are available on request from the corresponding author.

## Supplementary Materials

The following are available online at https://www.mdpi.com/article/10.3390/polym13234084/s1, Figure S1: Representative differential scanning calorimetry traces for synthesized materials in the dry state. Glass transition temperatures were taken as the half-height transition of the endothermic shift in the data. Figure S2: Representative images of lysates from coagulation time assay. Negative control contains empty tube with no samples.
