# Supplementary material for: Biostable Shape Memory Polymer Foams for Smart Biomaterial Applications
Source: Polymers (Basel). 2021 Nov 24;13(23):4084. doi: 10.3390/polym13234084 (PMC8658902; doi:10.3390/polym13234084)
Supplement: Supplementary file 1 [file polymers-13-04084-s001.zip › polymers-1461844-supplementary.pdf]

Supplementary Materials for:

# Biostable Shape Memory Polymer Foams for Smart Biomaterial Applications

**Anand Utpal Vakil, Natalie Marie Petryk, Ellen Shepherd and Mary Beth B. Monroe ***

Department of Biomedical and Chemical Engineering, Syracuse Biomaterials Institute, and BioInspired Syracuse: Institute for Material and Living Systems, Syracuse University, Syracuse, NY 13244, USA; auvakil@syr.edu (A.U.V.); nmpetryk@syr.edu (N.M.P.); eshepher@syr.edu (E.S.)

* Correspondence: mbmonroe@syr.edu; Tel.: +1-315-443-3323

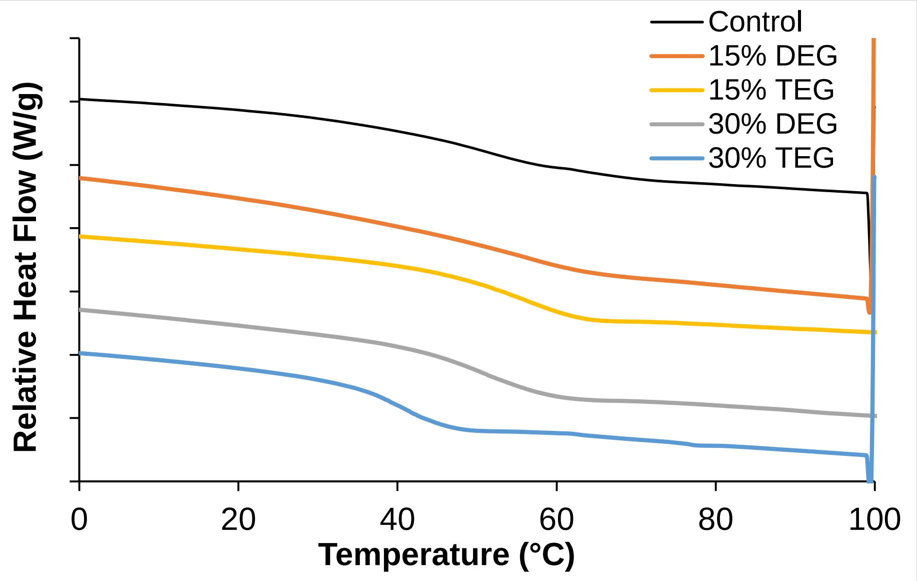


**Figure S1.** Representative differential scanning calorimetry traces for synthesized materials in the dry state. Glass transition temperatures were taken as the half-height transition of the endothermic shift in the data.

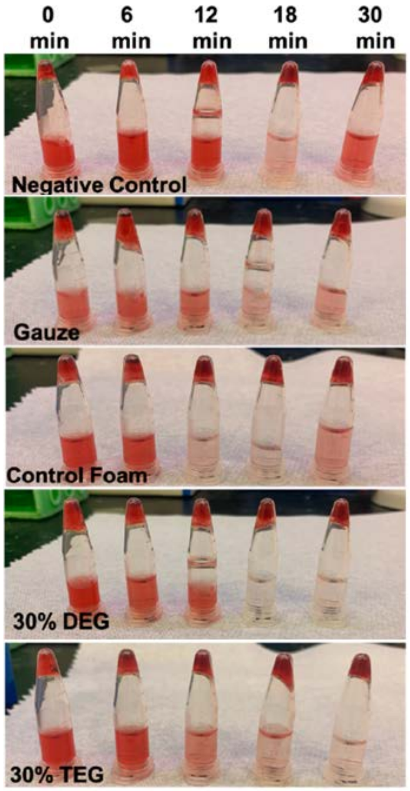


**Figure S2.** Representative images of lysates from coagulation time assay. Negative control contains empty tube with no samples.
